# The cepacian-like exopolysaccharide of *Paraburkholderia ultramafica* STM10279^T^ enhances growth and metal adaptation of *Tetraria comosa* on New Caledonian ultramafic soil

**DOI:** 10.3389/fpls.2024.1349724

**Published:** 2024-06-05

**Authors:** Alexandre Bourles, Guillaume Pierre, Hamid Amir, Alizée Le Floc’h, Eleftherios Chalkiadakis, Valérie Médevielle, Philippe Jourand, Philippe Michaud, Valérie Burtet-Sarramégna, Linda Guentas

**Affiliations:** ^1^ Institut de Sciences Exactes et Appliquées, Université de la Nouvelle-Calédonie, Noumea, New Caledonia; ^2^ Institut Pascal, Université Clermont Auvergne, Centre National de la Recherche Scientifique (CNRS), Clermont Auvergne Institut National Polytechnique (INP), Clermont-Ferrand, France; ^3^ Institut Universitaire de France (IUF), Paris, France; ^4^ BIOTECAL, Marine Biotechnology Company, Nouméa, New Caledonia; ^5^ Institute of Research for Development (IRD), UMR Entropie, Université de la Réunion, Saint Denis, France

**Keywords:** PGPR, exopolysaccharides, biostimulant, *Paraburkholderia*, plant metal alleviation, ultramafic soil, phosphate solubilization

## Abstract

*Paraburkholderia ultramafica* STM10279^T^ is a metal-tolerant rhizobacterium that promotes plant growth. It was isolated from the roots of *Tetraria arundinaceae*, a pioneer endemic tropical herb growing on ultramafic soils in New Caledonia. We have recently shown that the main mechanism of metal tolerance of *P. ultramafica* is related to the production of an acidic exopolysaccharide (EPS). To explore the potential role of this EPS in the plant’s environmental adaptation, we first elucidated its structure by employing a combination of chromatography and mass spectrometry techniques. These analyses revealed that the EPS is highly branched and composed of galactosyl (35.8%), glucosyl (33.2%), rhamnosyl (19.5%), mannosyl (7.2%), and glucuronosyl residues (4.4%), similar to the EPS of the *Burkholderia cepacia* complex known as cepacian. We subsequently conducted greenhouse experiments on *Tetraria comosa* plantlets inoculated with *P. ultramafica* or a solution of its EPS during transplanting onto ultramafic substrate. The data showed that the dry weight of *T. comosa* shoots was 2.5 times higher in the plants treated with the EPS compared to the unexposed plants. In addition, inductively coupled plasma–optical emission spectrometry (ICP-OES) analysis revealed that exposure to the EPS significantly increased Ca, Mg, K, and P uptake as well as K content in roots. *In vitro* experiments using the Pikovskaya method showed that the EPS was able to solubilize phosphorus. Consistent with the retention of metals in roots and a reduction in shoots, our data revealed a significant decrease in metal translocation factors (TFs) in the plants inoculated with the EPS. These results suggest a beneficial effect of the rhizobacterial EPS on plant growth and abiotic stress mitigation. In addition, the data suggest that the reduced levels of trace metals in plants exposed to *P. ultramafica* STM10279^T^ are due to metal chelation by the EPS. Further investigations are needed to firmly demonstrate whether this EPS could be used as a biostimulant for plant growth and adaptation to ultramafic soils.

## 1 Introduction

Plant-associated microorganisms are adapted to a wide range of environments, and most have been shown to improve plant growth or health (de la Fuente Cantó et al., 2020). Among these, plant growth-promoting rhizobacteria (PGPRs) can directly enhance plant growth by producing phytohormones and increasing nutrient availability in the rhizosphere (Khatoon et al., 2020). PGPRs are also known to increase tolerance to biotic and abiotic stresses (Zahedi, 2021). Many PGPRs secrete mixtures of exopolysaccharides (EPSs) of high molecular weight in response to physiological stress (Morcillo and Manzanera, 2021). EPS plays an important role in cell protection and bacterial adhesion to solid surfaces (i.e., biofilm formation) and is involved in cell–cell interactions (Netrusov et al., 2023). Recently, Naseem et al. (2018) reviewed the role of EPS-producing rhizobacteria on drought tolerance, plant growth, root and shoot biomass, and the physico-chemical properties of soil. In addition, it has been shown that some EPSs bind metals in soil (Prabhu et al., 2019), which affects the solubility of metal phosphates and plant nutrition (Kumar and Shastri, 2017). Moreover, metal-resistant PGPRs have been shown to mitigate metal stress in plants by reducing the bioavailability of trace metals through complexation by their secreted EPSs (Gupta and Diwan, 2017; Etesami, 2018). This process is of particularly high relevance to the ultramafic soils of New Caledonia where PGP bacteria play a crucial role in providing metal resistance to endemic plants, enabling their survival and growth in soils that would otherwise be inhospitable (Gonin et al., 2013; Guentas et al., 2016; Bourles et al., 2019; Bourles et al., 2020a).

New Caledonia is a unique biodiversity hotspot (Norman, 2003), with one-third of the main island covered by ultramafic soils. These soils, also known as serpentine soils, result from the weathering and pedogenesis of ultramafic rocks. They are characterized by i) the dominance of iron oxides with prominent levels of potentially toxic metals such as Ni, Co, Cr, and Mn; ii) low levels of key plant nutrients such as N, P, and K; and iii) a highly unbalanced Ca/Mg ratio (Brooks, 1987). Edaphic conditions led to the development of a specific flora characterized by 96.7% endemism (Isnard et al., 2016). In these extreme conditions, plants have developed several adaptive mechanisms, including i) slow growth, ii) the ability to limit Mg uptake, and iii) strategies to tolerate relatively high metal concentrations (Kazakou et al., 2008; Jaffré, 2023).

Ultramafic environments are exploited for economically valuable ores such as Ni, Co, and Cr (Pascal et al., 2008), and several strategies are used for ecological restoration (Losfeld et al., 2015). Recent studies have shown that the use of microorganisms appears to be a promising approach, regardless of the richness and phylogenetic diversity of the bacterial species found in New Caledonian ultramafic soils (Chaintreuil et al., 2007; Herrera et al., 2007; Klonowska et al., 2012; Gonin et al., 2013; Bourles et al., 2019; Bourles et al., 2020a).

Recently, we investigated the mechanisms underlying stress metal adaptation of the PGPR species *Paraburkholderia ultramafica* STM10279^T^. This bacterium was isolated from the roots of *Tetraria arundinaceae* (Cyperaceae), a pioneer species growing on ultramafic soils in New Caledonia (Guentas et al., 2016; Bourles et al., 2020b). We proposed that the EPS produced by this species may be responsible for the high tolerance of *P. ultramafica* STM10279^T^ to the extreme edaphic constraints of ultramafic soils (Bourles et al., 2020b) by binding metal cations. EPS production has been reported in the genus *Paraburkholderia* (Achouak et al., 1999; Serrato et al., 2006; Silipo et al., 2008; Suárez-Moreno et al., 2012), and the importance of the corresponding EPS in plant adaptation has been reviewed (Ferreira et al., 2011). Notably, EPS has been shown to play a role in plant–bacteria interactions and tolerance to environmental stress (Liu et al., 2020; Fu and Yan, 2023).

Given the potential role of the EPS of *P. ultramafica* STM10279^T^ in plant growth, we investigated the effects of EPS inoculation on the growth of *Tetraria comosa*. *T. comosa* ex *Costularia comosa* (Larridon et al., 2018) is a pioneer herbaceous plant belonging to the Cyperaceae family, the dominant family of herbaceous strata in New Caledonian ultramafic soils. *T. comosa* is used for restoration programs (Wulff et al., 2010), but it is characterized by slow growth. We hypothesize that EPS is involved in the interaction of *P. ultramafica* STM10279^T^ with plant tissues, which is also expected to occur under edaphic constraints, thereby conferring metal resistance to endemic plant hosts. Our data suggest that plant inoculation with the EPS produced by *P. ultramafica* STM10279^T^ offers significant advantages over inoculation with intact bacterial cells.

## 2 Materials and methods

### 2.1 *Paraburkholderia* strain

Plant sampling and isolation of the bacterial strains used in this work were as previously described (Gonin et al., 2013). The site used for this purpose was located in a ligno-herbaceous scrubland ecosystem on ultramafic cambisol at an altitude of 534 m. The geochemical soil characteristics are reported **in Supplementary Table S1**. The characterization of *P. ultramafica* STM10279^T^, including morphological, biochemical, and molecular traits, was performed as previously reported (Guentas et al., 2016). *P. ultramafica* STM10279^T^ isolated from ultramafic soils tolerates edaphic constraints such as low pH (range 4–8), unbalanced Ca/Mg ratios (1/19), and high concentrations of trace metals. *P. ultramafica* STM10279^T^ was also shown to exhibit specific properties relevant to the promotion of plant growth, such as the production of the phytohormones ACC and IAA, NH_3_, and siderophores. All details concerning its tolerance to edaphic constraints and PGP activity are reported in **Supplementary Table S1**.

### 2.2 Preparation of the bacterial inoculum

A primary culture of bacteria was established by inoculating 10 mL of Lysogenic Broth Lennox medium (LB; tryptone: 10 g/L, yeast extract: 5 g/L, and NaCl: 5 g/L; CONDA, Madrid, Spain) with one LB plate agar colony and incubating the culture at 28°C under orbital shaking at 125 rpm for 48 h. The primary culture was then used to inoculate 100 mL of LB medium at OD_600_ 0.1, and the culture was incubated for 72 h in the above conditions. The cells were then pelleted by a 20-min centrifugation at 5,000 *g* (Sigma, 2K15) and washed twice with sterile deionized water. The pellets were resuspended in sterile deionized water to reach an OD_600_ of 0.5 corresponding to a bacterial concentration of 10^7^ to 10^8^ CFU/mL, as suggested by Larcher et al. (2003). The cell suspensions were further diluted and plated on LB agar plates (three replicates) to obtain isolated colonies, and the cultures were incubated at 28°C.

### 2.3 EPS production and purification

The EPS of *P. ultramafica* STM10279^T^ was produced as described by Bourles et al. (2020b). To this end, 96-h cultures were grown at 28°C under 125 rpm orbital shaking in minimum broth medium (MB; KH_2_PO_4_ ·3H_2_O, 0.66 g/L; NaCl, 50 mg/L; MgSO_4_ ·7 H_2_O,10 mg/L; FeCl_3,_ 4 mg/L; CaCl_2,_40 mg/L) supplemented with glucose (30 g/L). Eight liters of LB was inoculated using a 48-h grown culture (800 mL; 28°C, 125 rpm orbital shaking) to produce enough biomass. After 72 h of incubation (28°C, 125 rpm orbital shaking), the biomass was recovered by centrifugation at 9,000 rpm for 25 min (Sorvall RC5C Centrifuge, DuPont, Wilmington, DE, USA) and washed once with sterile deionized water. Cells were transferred at 28°C in 10-L reactors containing 8 L of MB medium supplemented with glucose (3% w:v). The bacteria were removed from the medium by centrifugation at 9,000 rpm for 25 min. The supernatants were collected, and the EPS was purified by ultrafiltration on a 100,000 cut-off Sartorius nominal molecular weight cutoff (NMWCO) membrane and immediately freeze-dried. The EPS powders were collected and used for structural analyses and to prepare plant inoculum. In the latter case, the EPS was solubilized in sterile water at a concentration of 10 mg/mL.

### 2.4 EPS chemical analysis

#### 2.4.1 Polymer hydrolysis

Ten milligrams of polysaccharides was hydrolyzed in trifluoroacetic acid (TFA) (1 mL; 2 M) for 90 min at 120°C under gentle agitation (600 rpm).

#### 2.4.2 HPAEC-PAD analysis

The hydrolyzed sample (from 2.4.1) was neutralized using a 33% NH_3_ solution, centrifuged at 4°C (13,000 × *g*; 15 min, room temperature), diluted (1/10, 1/100, and 1/1,000), and filtered on 0.22-µm filters. Twenty-five microliters was injected on a Carbopac PA-1 column (Dionex Corporation, Sunnyvale, CA, USA; 4 × 250 mm) equipped with a pre-column (Dionex Corporation, 4 × 50 mm). The elution was performed at a flow rate of 1 mL/min using an isocratic gradient of NaOH (18 mM) for 30 min followed by a linear gradient of sodium acetate (NaOAc; from 0 to 1 M) in NaOH (200 mM) for 20 min. A pulsed amperometric detector was used for the analysis (Dionex Corporation, ICS 3000). The acquisition and processing software used was Chromeleon version 6.8.

#### 2.4.3 GC/MS-EI analysis

The EPS hydrolysate (from 2.4.1) was dried under nitrogen, washed twice with methanol (1 mL), and dried again. The derivatization was carried out as described by Pierre et al. (2012); Pierre et al. (2014) using BSTFA : TMCS (99:1) (2 h, room temperature, 600 rpm). The solvent was then evaporated under nitrogen, and the trimethylsilyl-*O*-glycosides were resuspended into dichloromethane (10 g/L) and diluted prior to analysis. Standard monosaccharides (Man, Glc, Ara, Rha, Rib, Fuc, Xyl, Fruc, ManA, GalA, and GlcA) were prepared under the same experimental conditions. The analyses were performed by gas chromatography/mass spectrometry–electron ionization (GC/MS-EI) using an Agilent 6890 Series GC System coupled to an Agilent 5973 Network Mass Selective Detector. The derivatives were injected on an OPTIMA-1MS (30 m, 0.32 mm, 0.25 μm) from Macherey-Nagel (Düren, Germany) with a helium flow rate of 2.3 mL/min. The helium pressure was set at 8.8 psi and the split ratio at 50:1. The following temperature program was used for the separation: 8°C/min to reach 100°C, 3 min at 100°C, 8°C/min to 200°C and 200°C for 1 min, and 5°C/min to >215°C (runtime 19 min 50 s). The ionization was carried out by EI (70 eV), with the trap temperature fixed at 150°C and the target ion set at 40–800 *m*/*z*. The injector temperature was 250°C.

### 2.5 Glycosidic linkage analysis

Pellets of solid NaOH were crushed in dimethyl sulfoxide (DMSO) as described by Ciucanu and Kerek (1984). Methylation was performed by a method adapted from Peña et al. (2012). Briefly, 2 mg of *P. ultramafica* EPS was resuspended in DMSO (200 to 500 μL), methylated using iodomethane, and hydrolyzed in TFA (2 M, 90 min, 120°C). The methylated monosaccharides were converted into partially *O*-methylated alditol acetates (PMAAs) using sodium borodeuteride and ethyl acetate. PMAAs were finally solubilized in dichloromethane (200 μL) and analyzed by GC/MS-EI using the same conditions and apparatus as described above.

### 2.6 Phosphate solubilization by the *P. ultramafica* EPS

Phosphate solubilization by *P. ultramafica* EPS was evaluated *in vitro* using the EPS alone or the EPS in citric acid as suggested by Yi et al. (2008). The freeze-dried EPS was sterilized by cold ethanol, centrifuged, and freeze-dried again. A solution of 10 mg of the EPS in 10 mL sterile deionized water and a stock solution of citric acid consisting of 19.2 mg in 10 mL sterile H_2_O were prepared. One milliliter of the EPS solution and 10 µL of citric acid solution were mixed and freeze-dried prior to the addition of 1 mL of Pikovskaya medium (PVK) (yeast extract: 0.5 g/L; glucose: 10 g/L; Ca_3_(PO_4_)_2_: 5 g/L; MgSO_4_, H_2_O: 0.1 g/L; MnSO_4_, H_2_O: 0.002; KCl: 0.2 g/L; NH_4_SO_4_: 0.5 g/L; FeSO_4_, 7H_2_O: 0.002 g/L; NaCl: 0.2 g/L). The PVK medium and a KH_2_PO_4_ solution (2 mg/L) were used as negative and positive controls, respectively. The mixtures were incubated at 28°C under lateral agitation (30 rpm). After 48 h, 5 mL H_2_Od, 1 mL chloromolybdic acid, 1 mL hydroquinone (10 g/L), and 1 mL sodium sulfite (200 g/L) were added. The appearance of a blue color indicates a positive result, i.e., phosphate solubilization (Pikovskaya, 1948). The concentration of the solubilized phosphate was estimated using a standard curve of KH_2_PO_4_ (**Supplementary Figure S1**; dilutions: 25, 50, 100, 150, 200, and 250 mg/L of KH_2_PO_4_; λ = 700 nm).

### 2.7 Greenhouse experiments

#### 2.7.1 Substrate preparation

The substrate used was a mixture of i) 2-mm sieved colluvial lateritic soil (ferralsol) sampled from the Plum area in New Caledonia (22°16′59″S, 166°39′12″E) with the following characteristics: coarse sand, 39.4%; fine sand, 22.1%; silt-clay, 37.2%; pH H_2_O, 5.9; pH KCl, 5.6; total C, 42.1 g/kg; total N, 2.2 g/kg; total P, 147 mg/kg; available P (Mehlich), 3 mg/kg; total Ca, 1.06 g/kg; total Mg, 5.08 g/kg; Ca/Mg, 0.207; and ii) commercial compost (4:1 v/v). The composition of commercial compost was as follows: N, 1.7 mg/g; P_total_, 150 mg/kg; P_Olsen_, 7 mg/kg; K, 139 mg/kg (Terreau universel, Agrofino, France). Metal contents of the ferralsol were as follows: Co, 0.87 g/kg; Cr, 22 g/kg; Fe, 348 g/kg; Mn, 10 g/kg; and Ni, 5.6 g/kg. Available metal extracted by dimethylene triaminopentaacetic acid (DTPA) were as follows: Co_DTPA_, 68 mg/kg; Cr_DTPA_, 0.17 mg/kg; Fe_DTPA_, 71 mg/kg; Mn_DTPA_, 1.1 g/kg; and Ni_DTPA_, 130 mg/kg. The addition of this commercial compost to the substrate for the greenhouse experiments was necessary because the growth of the plantlets of *T. comosa* is known to be inhibited in pure lateritic topsoil (Lagrange et al., 2011). The soil/compost mixture was autoclaved three times at 120°C for 1 h, with an interval of 24 h, to eliminate the soil’s original microorganisms.

#### 2.7.2 Plant production in nursery and plant inoculation/amendment

The seeds of *T. comosa* were provided by SIRAS Pacifique (Nouméa, New Caledonia). Germination was carried out on the soil/compost mixture described above. After 4 months, two- or three-leaf stage *T. comosa* plantlets were harvested as indicated elsewhere (https://www.agripedia.nc/sites/default/files/pdf/fiche_costularia-comosa.pdf) and washed with sterile water to remove any contaminants. The seedlings were then transplanted into 1-L plastic containers filled with approximately 600 g of the soil/compost mixture described above.

A comparison was made between non-inoculated plants, plants inoculated with *P. ultramafica* STM10279^T^, and plants exposed to the EPS produced by *P. ultramafica* STM10279^T^. A 5-mL bacterial suspension of 10^7^ and 10^8^ CFU/mL (corresponding to a 0.5 OD_600_) or EPS solution (10 mg/mL) or sterilized water (control) was applied once on the roots of each plantlet at transplanting (15 plants per inoculation treatment), and the plants were grown under greenhouse conditions for 8 months as described elsewhere (Lagrange et al., 2011). To control water supply and better reproduce environmental conditions, watering (100 mL) was manually conducted every 2 days. Plant growth was assessed over time by measuring the size of the largest leaf every 2 months. After harvesting the plants, the soil was removed from the roots by extensive washing, the shoots and roots were separated, and their mass was measured after drying at 60°C for 72 h (Bashan et al., 2017).

#### 2.7.3 Chemical analysis of plants

Plant tissue samples were pooled in groups of three replicates and ground to powder. The Ca, Mg, Na, K, P, Co, Cr, Fe, Mn, and Ni contents were measured in shoots and roots by inductively coupled plasma–optical emission spectrometry (ICP-OES; Varian®, Varian 730-ES, Palo Alto, CA, USA) after hydrolysis with a mixture of HNO_3_ (69%) and H_2_SO_4_ (37%) (4:1, v/v) at the “Laboratoire des Moyens Analytiques” (LAMA-US IMAGO-IRD, New Caledonia). The concentration of the different elements in the plant tissues was used to estimate the percentage of gain or loss in response to bacterial inoculation, as well as the translocation factor (TF). The gain or loss of a trace metal was calculated as [(Concentration in the tissue of inoculated plant − Concentration in the tissue of control plant) * 100)/Concentration in the tissue of control plant] (Gonin et al., 2013). The TF was calculated as [Concentration in the shoots of inoculated plant/Concentration in the roots of inoculated plant] (Galal and Sheata, 2015).

#### 2.7.4 Data and statistical analyses

All statistical analyses were performed using the R software version 3.3.1 (R Core Team, 2021) (version 1.2–4, available at http://CRAN.R-project.org/package=agricolae). All data were analyzed using ANOVA parametric tests, followed by Tukey’s honestly significant difference (HSD) test (p < 0.05) or with non-parametric tests (Kruskal–Wallis test followed by Fisher’s least significant difference (LSD) test (p < 0.05) when data did not follow the assumptions of a parametric test. Methods were applied independently for each plant species and each variable. For the greenhouse experiments, a principal component analysis (PCA) was carried out followed by a hierarchical clustering on principal components (HCPC), which allowed classification of the treatments according to the chosen variables.

## 3 Results

### 3.1 Chemical composition of the EPS of *P. ultramafica* STM10279^T^

The chemical composition of the EPS is shown in **Table 1**. High-performance anion-exchange chromatography (HPAEC) analysis showed the presence of galactosyl (Gal; 35.8%), glucosyl (Glc; 33.2%), rhamnosyl (Rha; 19.5%), mannosyl (Man; 7.2%), and glucuronosyl (GlcA; 4.4%) residues. The composition was verified by GC/MS analysis of *O*-trimethylsilylated derivatives, which indicated the following molar ratios (%): Gal (30.6%), Rha (25.0%), Glc (22.9%), Man (12.1%), and GlcA (9.4%).

**Table 1:** Biochemical and monosaccharide composition of the EPS produced by *Paraburkholderia ultramafica* STM10279^T^.

Sample	Production yield* mg EPS/L	Total carbohydrates*	Protein*	C/P*	Neutral sugar*	Uronic acid*	Monosaccharides (mol%) ^a^				
*P. ultramafica* STM10279^T^ EPS	140	61%	4%	15	22%	17%	Gal	Glc	Rha	GlcA	Man
							30.5	22.9	15	12.1	9.5

Gal, galactose; Glc, glucose; GlcA, glucuronic acid; Rha, rhamnose; EPS, exopolysaccharide.

*Previous results (Bourles et al., 2020b).

a Monosaccharide composition measured by gas chromatography/mass spectrometry–electron ionization (GC/MS-EI).

The data obtained from the GC/MS analysis of the PMAAs and corresponding glycosidic linkages are summarized in **Table 2**. The results show the presence of terminal Gal*p* (34.4%), (1,3)-Glc*p* (14.1%), (1,2,3)-Glc*p* (10.3%), (1,2,3)-Rha*p* (8.4%), (1,2)-Rha*p* (6.6%), (1,3)-Man*p* (6.4%), (1,3,6)-Man*p* (5.8%), and terminal Glc*p* (2.5%). These assessments were in keeping with the monosaccharide composition obtained after trimethylsilyl (TMS) derivatization (**Table 1**), except for Rha (15% *versus* 19.5%–25%). The GC/MS column and temperature program used for methylation analysis of the EPS did not allow the separation of Glc*p* from GlcA*p*. However, with the assistance of TMS analysis, we were able to estimate that 11.6% of the (1,2,3,4)-linked Glc*p* was in fact assignable to GlcA*p*. The proportion of Gal (34.4%):Glc (26.9%):Rha (15%):Man (12.1%):GlcA (11.6%) was close to 3:2.3:1.3:1:1. Furthermore, terminal and branched total residues % were close (36.98% and 36.02%, respectively).

**Table 2:** Partially methylated alditol acetates (PMAAs) and glycosidic linkages of the EPS produced by Paraburkholderia ultramafica STM10279T.

Partially *O*-methylated alditol acetates^a^	%^b^	Linkage type^c^
2,3,4,6-Me_4_-Gal	34.4	Gal*p*-(1→
*Total*	**34.4**	
2,3,4,6-Me_4_-Glc	2.48	Glc*p*-(1→
2,4,6-Me_3_-Glc	14.1	→3)-Glc*p*-(1→
4,6-Me_2_-Glc	10.3	→2,3)-Glc*p*-(1→
6-Me-Glc	11.6	→2,3,4)-Glc*p*-(1→
*Total^d^*	**38.5**	
3,4-Me_2_-Rha	6.58	→2)-Rha*p*-(1→
4-Me-Rha	8.37	→2,3)-Rha*p*-(1→
*Total*	**15.0**	
2,4,6-Me_3_-Man	6.35	→3)-Man*p*-(1→
2,4-Me_2_-Man	5.75	→3,6)-Man*p*-(1→
*Total*	**12.1**	

All analyses were run in duplicate, and the relative standard deviations were less than 5%.

a 2,4,6-Me_3_-Glc = 2,4,6-tri-O-methyl-glucitol-acetate, etc.

b % of peak area of O-methylated alditol acetates relative to total area, determined by gas chromatography/mass spectrometry (GC/MS).

c Based on derived O-methylated alditol acetates.

d Corresponded for both Glcp and GlcAp.

The main backbone (48.1%) of the EPS could thus be composed of (1,3)-linked Glc*p* (and/or GlcA*p*) and (1,3)-linked Man*p*. The presence of primary branched sugar residues (1,2,3)-linked Glc*p* (10.3%), (1,3,6)-linked Man*p* (5.75%), and (1,2,3,4)-linked GlcA (11.6%) suggests that the main chain is highly branched (>60%) throughout the *O*-4 of (1,3)-linked GlcA*p*, probably by (1,2)-Rha*p*. The ramifications are probably composed of [1-Rha*p*-(2→1)-Gal*p*] substituted with putative Glc*p* through the *O*-3 position of the (1,2)-Rha*p*. The core-chain unit may be also substituted by Gal*p* through the *O*-2 position of (1,3)-linked Glc*p* or (1,2,3,4)-linked GlcA*p* and the *O*-6 positions of the (1,3)-linked Man*p*. The non-reducing ends in the EPS of *P. ultramafica* most likely correspond to t-Gal*p* (34.4%) and t-Glc*p* (2.48%).

### 3.2 Phosphorus solubilization

The experiment showed a phosphorus-solubilizing effect of the *P. ultramafica* STM10279^T^ EPS. A semi-quantitative assessment of phosphorus solubilization was made by measuring absorbance at 700 nm (**Supplementary Figure S1**; **Supplementary Table S2**). The results showed a positive effect of *P. ultramafica* STM10279^T^ EPS on phosphate solubilization, with an average solubilization of 74.9 ± 32.1 mg phosphorus (expressed in KH_2_PO_4_ equivalents) per 10 mg EPS (n = 3). The addition of citric acid was not accompanied by an increase in phosphate solubilization (**Supplementary Table S3**).

### 3.3 Plant growth, and shoot and root biomass

**Figure 1:**
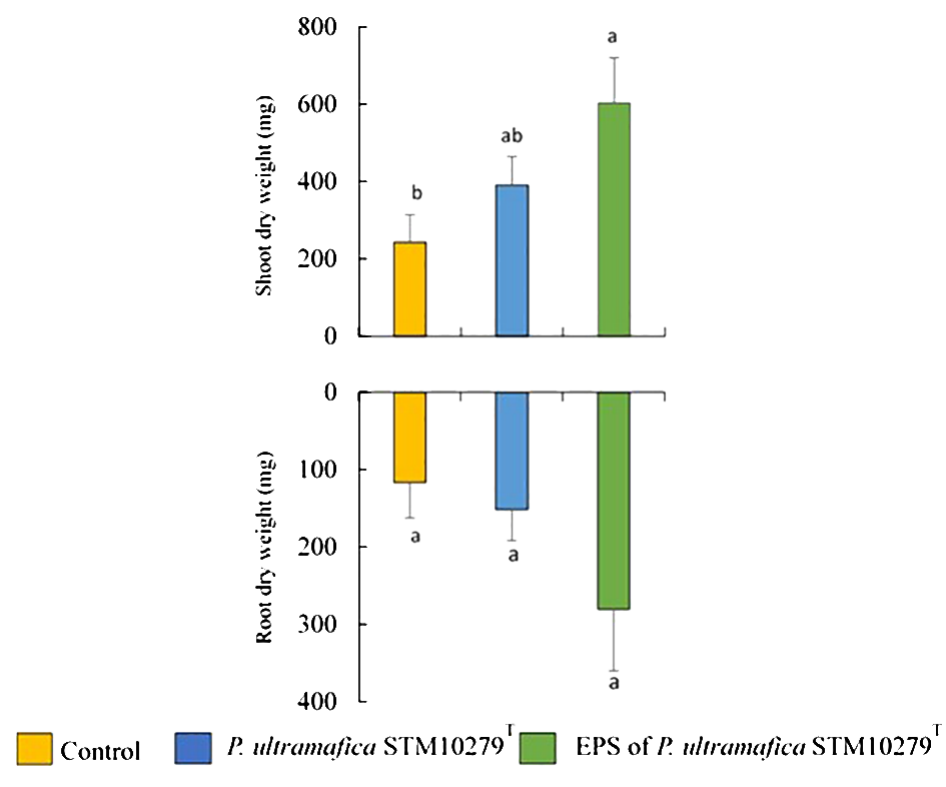
Influence of bacterial or exopolysaccharide (EPS) inoculation on *Tetraria comosa* on dry weight (mean values of height with standard errors). For each measure part of the plant, different letters above columns indicate significant differences at p < 0.05.

Photographs of *T. comosa* inoculated with bacteria or EPS, removed from pots after 8 months of growth in ultramafic soil, are shown in **Supplementary Figure S3**. Throughout the experimental period, no significant differences were observed between the different treatments. At the end of the experiment, *T. comosa* plants had grown to an average height of 16 ± 0.4 cm. The effect of bacterial and EPS inoculation on shoot and root dry weight is shown in **Figure 1**. The shoot dry weight of *T. comosa* inoculated with the EPS of *P. ultramafica* STM10279^T^ was the highest (549 ± 117 mg) with a significant difference compared to the control (2.5 times higher). With regard to the root, no significant differences were observed between the treatments. However, *T. comosa* plants inoculated with the *P. ultramafica* STM10279^T^ EPS also showed the highest value of root dry weight (281 ± 80 mg, 2.5 times higher than the control, **Supplementary Table S4)**.

### 3.4 Plant nutrient content and uptake

Inoculation of the *P. ultramafica* STM10279^T^ EPS (**Supplementary Figure S2**; **Supplementary Table S5**) significantly enhanced P concentration (455 ± 24 mg/kg; + 18%) in shoots compared to the control. In root tissues, K concentration was significantly higher in the plant inoculated with the EPS (6.8 ± 0.0 mg/kg; +13%) compared to the control. No significant differences were observed in either tissue for the Ca/Mg ratio (**Table 3**).

**Table 3:** Ca/Mg ratio in *Tetraria comosa* as a function of various treatments (mean ± SE, n = 3).

Plant Part	Treatment	Ca/Mg		
Shoot	Control	2.63	±	0.10a_1_^†,‡^
	*Paraburkholderia ultramafica* STM10279^T^	2.63	±	0.06a_1_
	EPS of *P. ultramafica* STM10279^T^	3.00	±	0.26a_1_
Root	Control	1.09	±	0.04a_2_
	*P. ultramafica* STM10279^T^	0.95	±	0.02a_2_
	EPS of *P. ultramafica* STM10279^T^	1.10	±	0.09a_2_

EPS, exopolysaccharide.

^†^Different letters in each row indicate significance at p < 0.05.

^‡^Same coefficient to letters refers to one plant part analysis.

**Figure 2:**
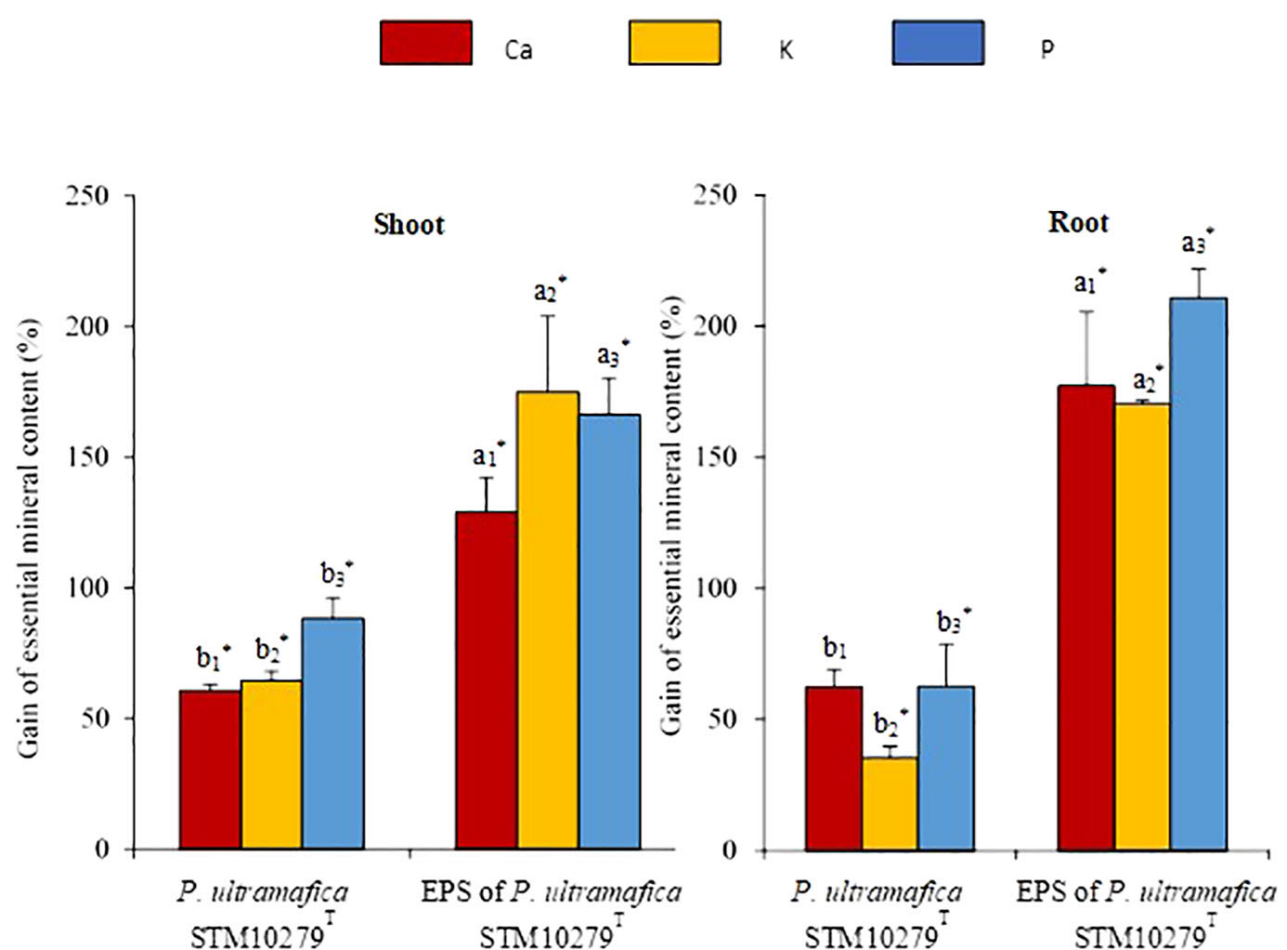
Effect of inoculation on essential mineral uptake of *Tetraria comosa* grown on ultramafic substrate. Relative variations of element content in shoots and roots are expressed as % of element content in dry mass of tissues of inoculated plants compared with controls. Bars represent means, and error bars represent standard deviation of means (n = 3). Different letters above columns indicate significant differences at p < 0.05. The same coefficient to letters refers to one element analysis. The letters and coefficients with an asterisk indicate significant differences with controls at p < 0.05.

Variations of the Ca, K, and P uptake in the roots and shoots of the *T. comosa* inoculated plants in comparison to the non-inoculated plants are presented in **Figure 2**, while full data are reported in **Supplementary Table S7**. Consistent with biomass increase, inoculation of the EPS (**Figure 2**; **Supplementary Table S6**) significantly enhanced Ca uptake (shoot: 2.4 mg/plant, 2.4 times higher; root: 0.5 mg/plant, 2.8 times higher), K uptake (shoot: 6.2 mg/plant, 2.7 times higher; root: 1.9 mg/plant, 2.7 times higher), and P. uptake (shoot: 249 µg/plant, 2.7 times higher; root: 94 µg/plant, 3.1 times higher) compared to the control.

### 3.5 Metal content

**Figure 3:**
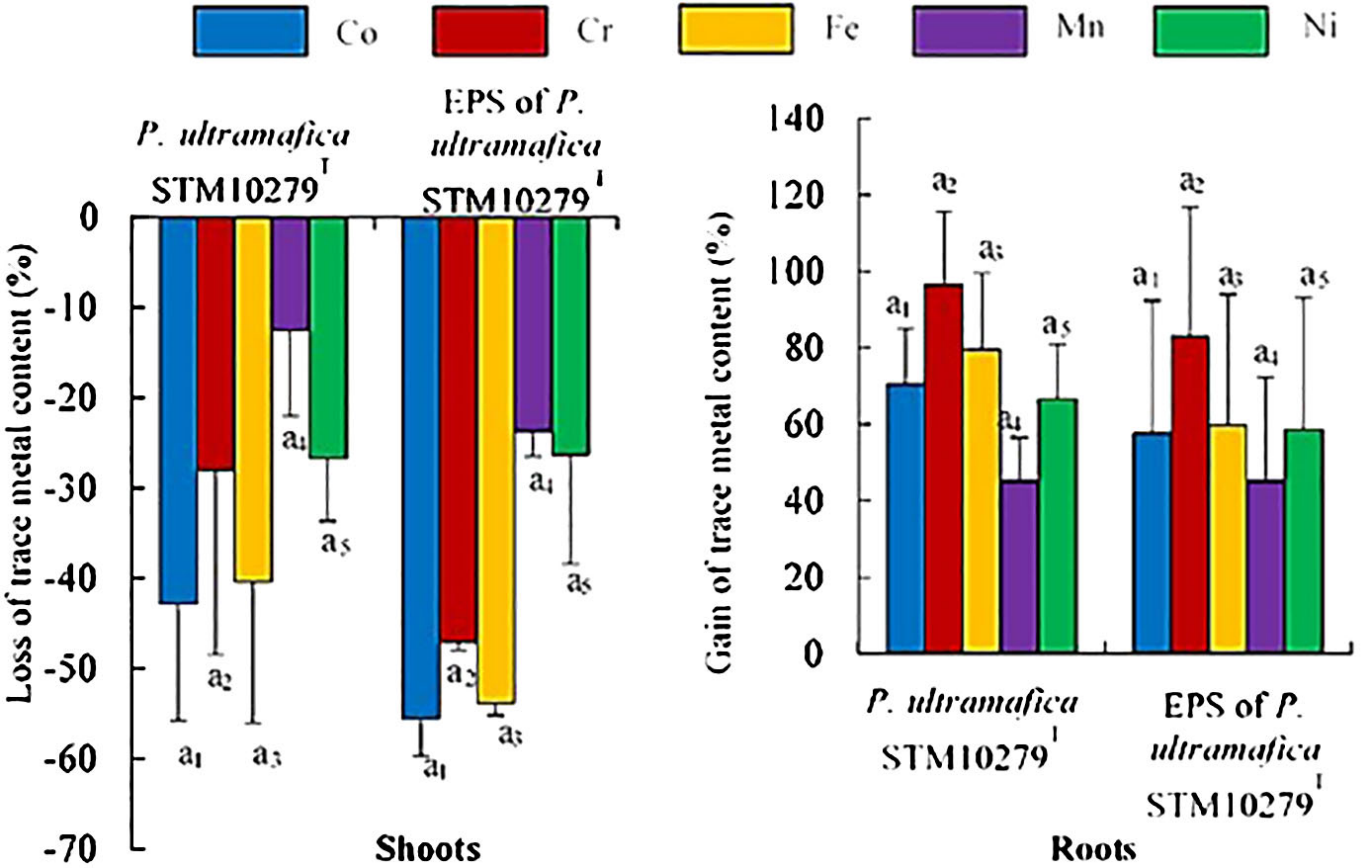
Effect of inoculation on essential mineral content of *Tetraria comosa* grown on ultramafic substrate. Relative variations of element content in shoots and roots are expressed as % of element content in dry mass of tissues of inoculated plants compared with controls. Bars represent means, and error bars represent the standard deviation of means (n = 3). Different letters above columns indicate significant differences at p < 0.05. The same coefficient to letters refers to one element analysis. The letters and coefficients with an asterisk indicate significant differences with controls at p < 0.05.

In shoot tissues, inoculation with *P. ultramafica* STM10279^T^ and exposure to the EPS led to a reduction in all trace metals, but no significant differences were observed compared to the control (**Figure 3**; **Supplementary Table S7**). In roots, an increase in trace metal content was observed with both inoculations, but also no significant differences were observed compared to the control. Metal translocations (**Table 4**) were significantly reduced with the inoculation of *P. ultramafica* STM10279^T^ and with the EPS, but significant differences were observed only for the translocation of Co, Cr, and Fe compared to the control. No significant differences were observed between plants inoculated with *P. ultramafica* STM10279^T^ or exposed to the *P. ultramafica* STM10279^T^ EPS.

**Table 4:** Trace metal translocation factor (TF) (mean ± SE, n = 3) as a function of various treatments.

Trace metal	Co			Cr			Fe			Mn			Ni		
Control	0.38	±	0.05a_1_^†,‡^	0.52	±	0.08a_2_	0.34	±	0.05a_3_	0.54	±	0.05a_4_	1.09	±	0.16a_5_
*Paraburkholderia ultramafica* STM10279^T^	0.15	±	0.03b_1_	0.21	±	0.05b_2_	0.13	±	0.02b_3_	0.4	±	0.02a_4_	0.56	±	0.02a_5_
EPS of *P. ultramafica* STM10279^T^	0.16	±	0.06b_1_	0.18	±	0.04b_2_	0.13	±	0.04b_3_	0.39	±	0.10a_4_	0.71	±	0.28a_5_

nd, not determined; EPS, exopolysaccharide.

^†^Different letters in each row indicate significant differences according to Tukey’s honestly significant difference (HSD) test or the least significant difference (LSD) Fisher’s post-hoc test (p < 0.05).

^‡^Same coefficient to letters refers to one data analysis of one TF.

### 3.6 PCA

**Figure 4:**
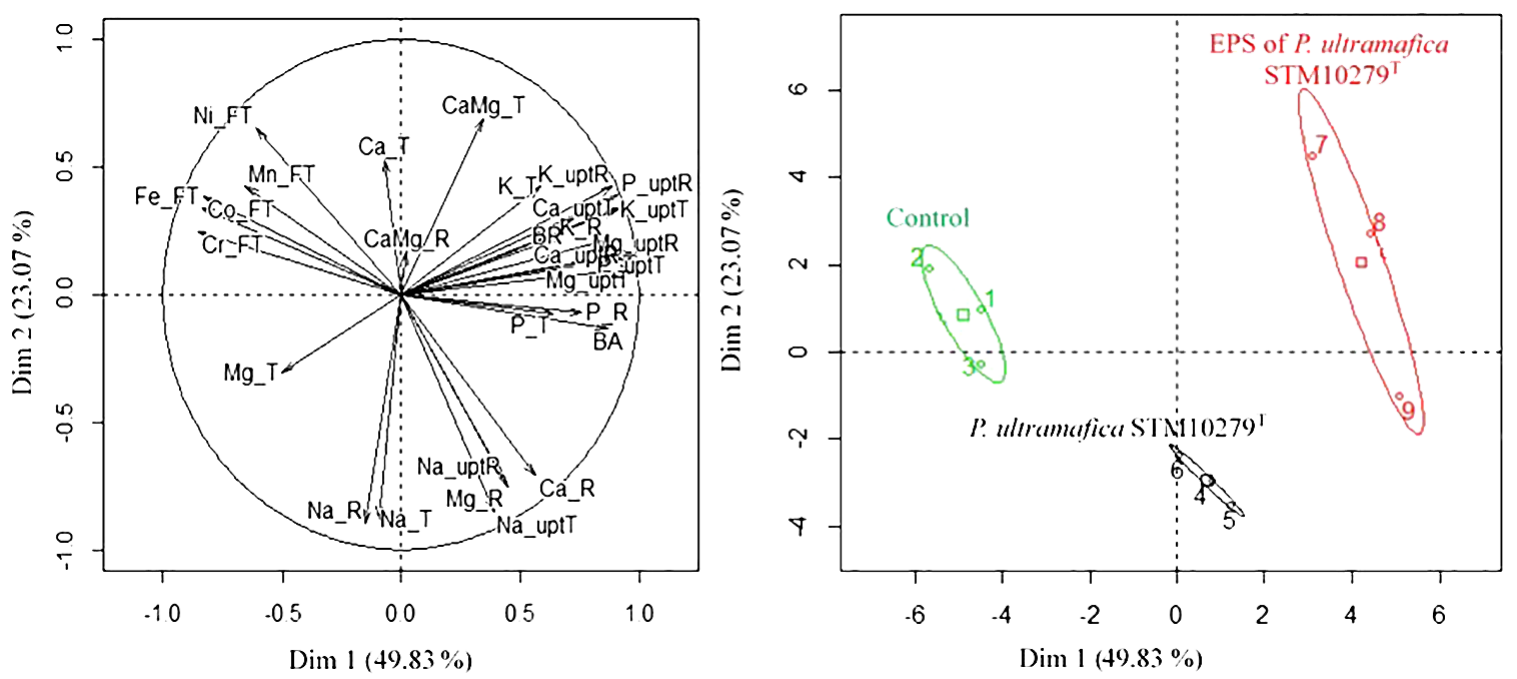
Principal component analysis (PCA) and hierarchical clustering on principal components (HCPC). PCA and HCPC were performed with 29 variables: biomass [dry weight tissue of shoots (BA) and roots (BR)], mineral and metal content in shoots (i.e., Ca_T) and roots (i.e., Ca._R) (Ca, Mg, K, Na, and P), metal translocation factor (i.e., Co_FT) (Co, Cr, Fe, Mn, and Ni), mineral element uptake in shoots (i.e., Ca_uptT) and roots (i.e., Ca_uptR) (Ca, Mg, Na, K, and P), and Ca/Mg ratio in shoots (CaMg_T) and roots (CaMg_R). Analyses were carried out using R software with the “FactoMineR” package. HCPC used Euclidean distances for calculating dissimilarities between observations and average method to define clusters.

A PCA was carried out to analyze the effect of the multiple variables arising from the experiments described above (**Figure 4**). The analysis showed that two principal components (PC1 and PC2) explained 72.9% of the total variation. The variables involved in the clustering of each group are presented in **Table 5**. Briefly, control plants are characterized by significantly higher i) translocation of Co, Cr, and Fe and by significantly lower (i) contents of Mg, P in the root; ii) uptake of P, K, Ca, and Mg in shoots; iii) uptake of P and Mg in roots; and iv) root biomass compared to the other treatments. *T. comosa* inoculated with *P. ultramafica* STM10279^T^ are characterized by significantly higher i) Na content in shoot and root, ii) Mg content in root, and iii) Na uptake in shoot compared to the other treatments. Inoculation of the *P. ultramafica* STM10279^T^ EPS significantly enhanced the uptake of Ca, Mg, K, and P and K contents in roots.

**Table 5:** Variables that defined each cluster (p < 0.05) using a principal component analysis (PCA) and hierarchical clustering on principal components (HCPC).

Cluster	Treatment		Variables
1	Control	+	Fe_FT, Cr_FT, Co_FT
		−	P_uptR, Mg_R, Ca_uptR, K_uptT, P_R, Ca_uptT, BA, Mg_uptR, P_uptT, Mg_uptT
2	*Paraburkholderia ultramafica* STM10279^T^	+	Mg_R, Na_R, Na_upT, Na_T
		−	
2	EPS of *P. ultramafica* STM10279^T^	+	Ca_uptT, K_uptT, Ca_uptR, Mg_uptT, Mg_uptT, P_uptR, K_uptR, K_R,P_uptT
		−	

PCA and HCPC were performed with 29 variables: biomass [dry weight tissue of shoots (BA) and roots (BR)], mineral and metal content in shoots (i.e., Ca_T) and roots (i.e., Ca._R) (Ca, Mg, K, Na, and P), metal translocation factor (i.e., Co_FT) (Co, Cr, Fe, Mn, and Ni), mineral element uptake in shoots (i.e., Ca_uptT) and roots (i.e., Ca_uptR) (Ca, Mg, Na, K, and P), and Ca/Mg ratio in shoots (CaMg_T) and roots (CaMg_R). Analyses were carried out using R software with the “FactoMineR” package. HCPC used Euclidean distances for calculating dissimilarities between observations and average method to define clusters.

EPS, exopolysaccharide.

## 4 Discussion

EPS plays important roles in plant–bacteria interaction and tolerance to environmental stresses (Bhagat et al., 2021) and has also been found to be important in facilitating plant–endophyte interactions, especially under stress conditions (Liu et al., 2017, 2020). A recent study by Fu and Yan (2023) has shown that EPS production is necessary for optimal colonization of the endophytic bacterium *Paraburkholderia phytofirmans* PsJN in plant tissues under drought-stress conditions. Given the numerous beneficial properties of EPS under varied stress conditions, as discussed in previous literature (Bhagat et al., 2021), our aim is to study the specific plant growth-promoting and metal resistance properties of the cepacian-like EPS produced by *P. ultramafica* STM10279^T^, a bacterium previously isolated in our laboratory (Bourles et al., 2020b).

### 4.1 *P. ultramafica* STM10279^T^ exopolysaccharide

The most common exopolysaccharide produced by *Burkholderia* gender is cepacian. Cepacian is a well-known acetylated EPS whose repeating unit consists of a heptasaccharide repeating unit comprising d-Glc, d-Rha, d-Man, d-Gal, and d-GlcA in a ratio of 1:1:1:3:1 (Cescutti et al., 2010). It has been found in different species belonging to environmental or clinical isolates (Ferreira et al., 2011). Ferreira et al. (2010) showed that the proteins involved in cepacian production are well conserved among the *Burkholderia* genus. External factors directly influence the type and number of EPS produced; indeed, some strains produced a single EPS, while others produced a mixture. Hallack et al. (2010) described the EPS “B” for *Paraburkholderia kururiensis*, a mixture of cepacian and an octosaccharide polymer. Bourles et al. (2020b) demonstrated the ability of *P. ultramafica* STM10279^T^ to produce an EPS when the strain was cultivated in a glucose-rich medium. The recovered EPS is composed of 17% uronic acid, and the carbohydrate-to-protein (C/P) ratio was almost 15, with values of 61% carbohydrate to 4% protein residues. Infrared spectroscopy analysis using a Frontier™ (PerkinElmer, Waltham, MA, USA) Fourier transform infrared (FT-IR) spectrometer revealed that the monosaccharides have pyranose rings and the presence of uronic acid groups. Bourles et al. (2020b) showed that the main mechanism involved in the Ni tolerance of the metal-resistant PGP *P. ultramafica* STM10279^T^ strain was related to the production of an acidic exopolysaccharide.

In this study, we examined the composition of the EPS of *P. ultramafica* STM10279^T^ and found that it is an anionic EPS containing glucuronic acid. Its structure is highly branched with over 60% branching. The structure is very similar to cepacian (Cérantola et al., 1999), but differs in two ways: first, there is a terminal glycosyl residue (2.48%) instead of the lateral chain throughout the *O*-3 position of the (1,2)-Rha*p* as in the structure of the EPS “B” of *P. kururiensis* (Hallack et al., 2010); second, the main chain of the EPS contains and unsubstituted (1,3)-linked Man*p* and is substituted throughout the *O*-2 position of the (1,3)-Glc*p* probably by Gal*p*. **Table 6** shows the hypothetical structural formula of the repeating unit of the EPS of *P. ultramafica* and a comparison with the structures of cepacian and EPS “B” of *P. kururiensis*.

**Table 6:** Hypothetic formula of the exopolysaccharide repeated units of the *Paraburkholderia ultramafica* STM10279^T^ and comparison with the closest EPS repeated unit described in the literature.

EPS/species	Structure	Composition: molecular ratio	Reference
Cepacian (PSII)/Bcc and non-Bcc	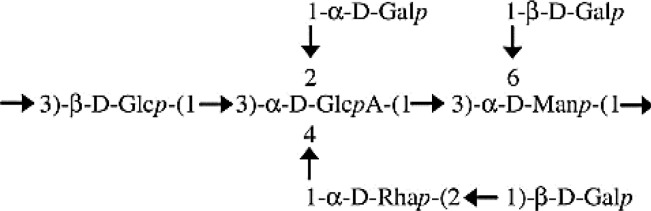	3 Gal*p* 1 Glc*p* 1 GlcA*p* 1 Rha*p* 1 Man*p*	Cérantola et al., 1999
EPS B/*Paraburkholderia kururiensis*	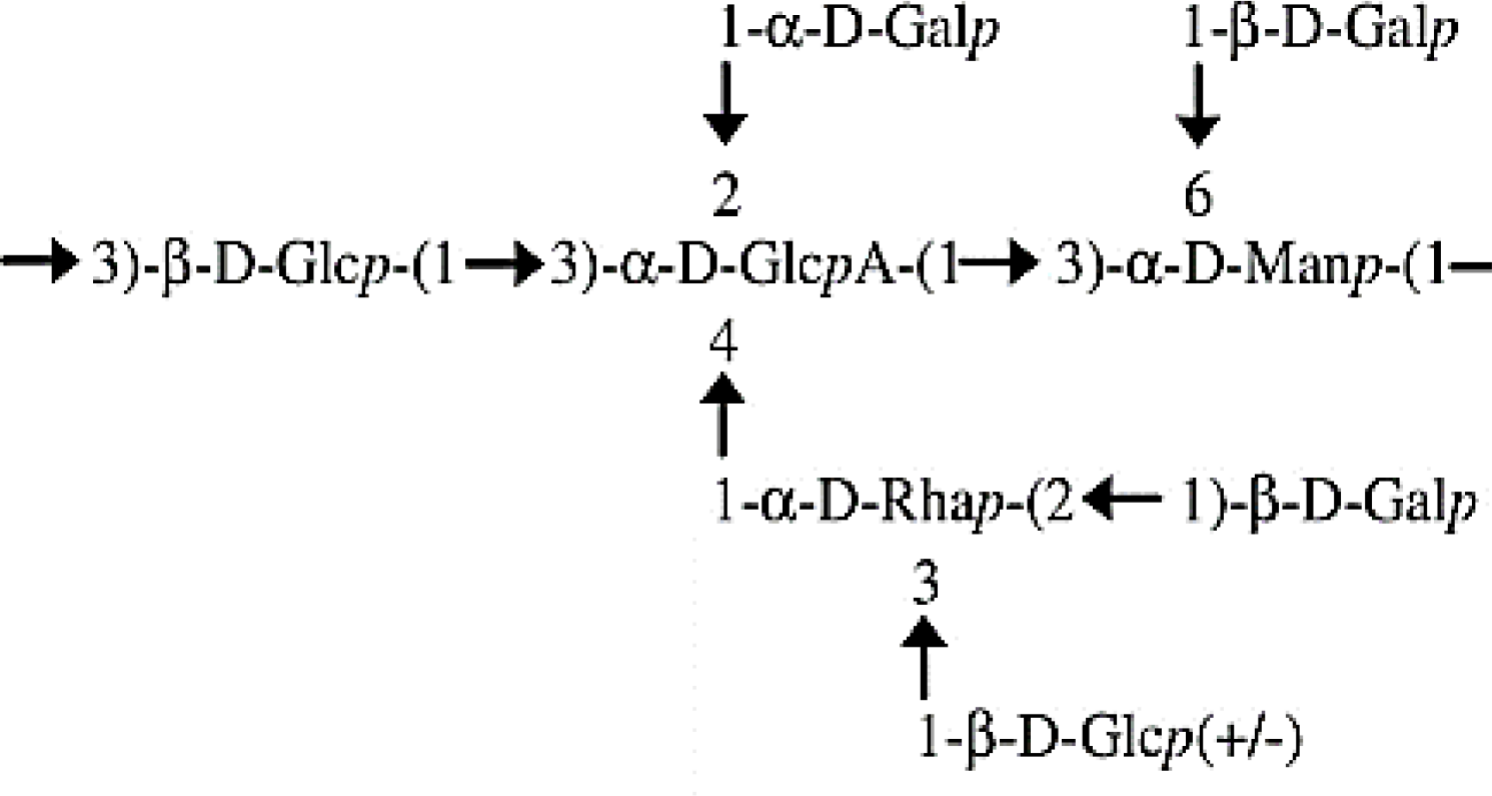	3 Gal*p* 1.5 Glc*p* 1 GlcA*p* 1 Rha*p* 1 Man*p*	Hallack et al., 2010
EPS of *P. ultramafica* STM10279^T^	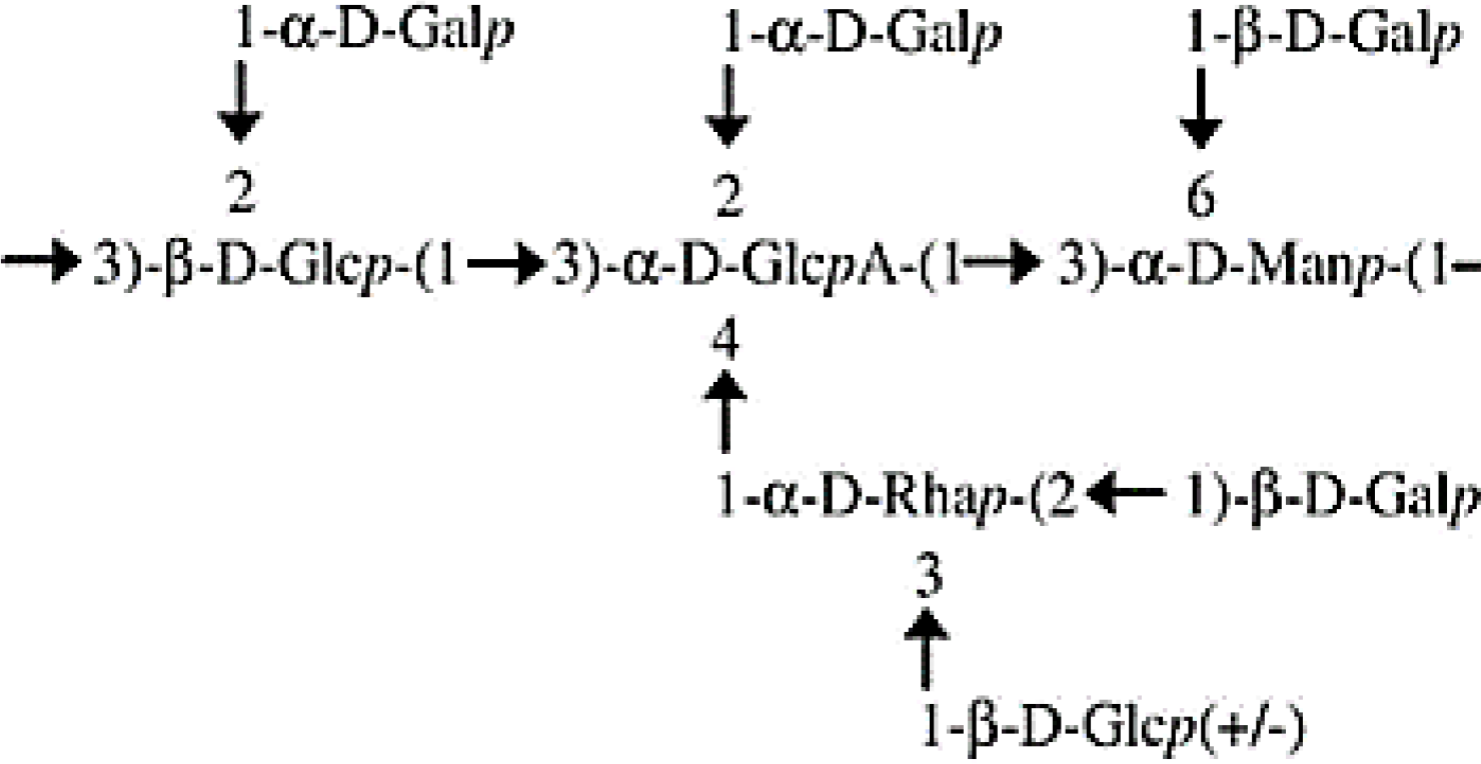	3 Gal*p* 2.3 Glc*p* 1 GlcA*p* 1.3 Rha*p* 1 Man*p*	This study

EPS, exopolysaccharide.

### 4.2 Effects *in planta* of *P. ultramafica* STM10279^T^ and its EPS

ETMs (trace metal elements), or essential trace metals, interact with plants through various metabolic and physiological pathways. When soils have high ETM concentrations, it can result in reduced shoot and root biomass. This can cause seedlings to have poorly developed root systems, which can make it difficult for them to absorb nutrients that are essential for proper plant development. In fact, seedlings growing on soils contaminated with ETMs may not be able to absorb the necessary nutrients (Hameed et al., 2015).

High concentrations of ETMs in the aerial part of the plant can have a negative effect on chlorophyll content and can inhibit the activity of certain enzymes, which can slow down metabolic pathways. While certain ETMs, such as Mn, Mo, and Ni, are essential for proper cell functioning, an excess of these metals can inhibit a large number of plant enzymes, particularly those involved in the Calvin cycle and chlorophyll biosynthesis. The real danger with most ETMs lies in their oxidation state and their ability to interact with the cellular components, leading to the formation of reactive oxygen species (ROS) that disrupt cellular metabolism. This can cause symptoms like chlorosis and necrosis, leading to a reduction in the plant’s growth and vitality and even death (Hossain et al., 2012; Evlard and Campanella, 2013). Several studies have been conducted to explore the impact of inoculating *P. ultramafica* STM10279^T^ on sorghum as well as on two native plants of New Caledonian ultramafic soils: *Metrosideros laurifolia* and *T. comosa* (Gonin et al., 2013; Gensous, 2014; Guentas et al., 2016; Bourles et al., 2020b). The primary effect of inoculating *P. ultramafica* STM10279^T^ was observed in the reduction of trace metals in tissues. Bourles et al. (2020b) proposed that this effect could be attributed to the bacteria’s ability to produce EPSs. EPS production can lead to biomineralization of metal ions, which can enhance metal stress tolerance in plants (Bhagat et al., 2021). Several studies have reported that EPS can bind with cationic ETMs owing to the presence of anionic functional groups, which results in the formation of EPS–metal complexes (Mishra et al., 2017). These complexes restrict the mobility of the metal ions to the plants (Bhagat et al., 2021). The functional groups, such as hydroxyl and carboxyl, present in the polysaccharides, play a crucial role in the metal binding process (Gupta and Diwan, 2017; Etesami, 2018). To test this hypothesis, we inoculated *T. comosa* with EPS produced by *P. ultramafica* STM10279^T^. To the best of our knowledge, this is the first study to demonstrate the effect of a microbial EPS on plant growth in ultramafic soils. Indeed, although the role of EPS in plant interactions is well established (Naseem et al., 2018), especially in *Burkholderia* species (Ferreira et al., 2011), no study has directly demonstrated the effects of EPS inoculation on host plants.

In our experiment where plants were inoculated with *P. ultramafica* STM10279^T^ or amended with its EPS, it was observed that metal translocation factors decreased significantly. This suggests that metals are immobilized at the root level and not translocated to the aerial parts. However, no significant differences in root physiology or shoot symptoms were observed. Plants amended with EPS of *P. ultramafica* STM10279^T^ had the highest values of shoot dry weight, indicating that supplementing the ETM roots with *P. ultramafica*’s EPS could improve the plant’s status. Additionally, metal accumulation at the root level corresponds to a phytostabilization process, which reduces the mobility and availability of contaminants in the soil (Alkorta et al., 2010). This helps to stabilize the contaminated site and prevent the dispersion of contaminants by erosion, reducing their impact on the food chain. Our experiment found higher concentrations of ETMs in the roots of inoculated plants compared to non-inoculated plants. However, a previous study by Bourles et al. (2020b) showed a significant decrease in metal concentrations in the roots after inoculation with *P. ultramafica* STM10279^T^. The difference in results could be due to the duration of the experiment (8 months in our study compared to the study of Bourles et al.). Despite the difference in results, we observed a similar effect in plants inoculated with *P. ultramafica* STM10279^T^ or amended with its EPS, which underlines the involvement of EPS in the alleviation of metals. This finding supports the hypothesis of Bourles et al.

Indeed, EPS is known to improve soil aggregation, water permeability, and nutrient uptake at the root level, as reported in studies by Vejan et al. (2016) and Upadhyay et al. (2012). Different watering methods were observed in various experiments, which could explain the varying effects seen with *P. ultramafica* STM10279^T^ inoculation. Rubin et al. (2017) demonstrated that PGPRs are more effective in mitigating water stress, while Bourles et al. (2020b) used an automatic watering system that irrigated the plants twice a day, but this method did not replicate the natural conditions (L'Huillier et al., 2010). In our study, watering was reduced to a manual process (100 mL) every 2 days. These differences highlight the fact that there are still parameters in the rhizosphere that remain to be discovered. Bourles et al. (2020b) found that the inoculation with *P. ultramafica* STM10279^T^ did not have a positive effect on the biomass of *T. comosa*; nor did it have any significant effects on plant nutrition. Our study, in contrast, showed that shoot and root dry weight increased when the plantlet was amended with *P. ultramafica* EPS, although this result was not statistically significant at the time of sampling. Our study also highlighted a significant effect of *P. ultramafica* EPS on trace metal contents compared to non-inoculated plants. However, the effect of metals does not explain the differences observed in growth. Bourles et al. (2020b) suggested that the lack of effect on growth could be explained by the lack of effect on concentrations of essential minerals.

One of the main nutrients limiting the plant growth is phosphorus. On ultramafic soils, the lack of essential plant nutrients is a major limitation for plants (Brooks, 1987). On ultramafic soils in New Caledonia, the amount of phosphorus is limiting for plant growth (L'Huillier et al., 2010). Phosphorus is adsorbed by the abundant iron and aluminum oxides and hydroxides found in these soils, making it unavailable to plants (Gobat et al., 2010). Plants use various strategies to acquire phosphorus from their surroundings, including modification of the root system (López-Bucio et al., 2003), establishment of mycorrhizal symbiosis (Amir and Ducousso, 2010), or interaction with phosphate-solubilizing bacteria (Gonin et al., 2013; Guentas et al., 2016).

Arbuscular mycorrhizal fungi (AMF) are known to enhance plant growth by improving mineral nutrition, particularly phosphorus and potassium (Smith and Read, 2008). The ability of some rhizobacteria to convert insoluble forms of phosphorus into soluble forms is a sought-after trait in PGPRs, particularly in ultramafic soils. The release of low-molecular-weight organic acids is the most common mechanism for P solubilization by phosphate-solubilizing bacteria (PSBs) (Goldstein, 1995). In fact, 33 out of 36 rhizobacterial strains isolated by Chen et al. (2006) from subtropical soils were found to excrete organic acid for P solubilization. Three of their P-solubilizing isolates did not produce organic acid, suggesting that another type of molecule is involved.

Yi et al. (2008) investigated the role of exopolysaccharides in the microbial-mediated solubilization of P. Bacterial strains belonging to the genera *Enterobacter*, *Arthrobacter*, and *Azotobacter* and having the ability to solubilize tri-calcium phosphate were used to evaluate the role of EPS in the solubilization of P. The authors demonstrated that EPS altered P solubilization homeostasis, favoring P dissolved by retaining free phosphorus in the medium, leading to a greater release of phosphorus from insoluble phosphate. In New Caledonia, particular attention has been paid to phosphorous in Cyperaceae (Lagrange, 2009; Lagrange et al., 2011, 2013; Gensous, 2014). Plants in the Cyperaceae family are generally considered to be non-mycorrhizal or very weakly mycorrhizal (Brundrett and Tedersoo, 2019). However, Lagrange et al. (2011) showed that mycorrhizal colonization is functional under greenhouse conditions in *T. comosa*, one of the most common sedges in the ultramafic maquis.

Bourles et al. (2020a) have demonstrated synergistic microbial interactions between *Curtobacterium citreum* BE strains, rhizobacteria, and AMF, particularly for P nutrition. *C. citreum BE* is a PSB capable of producing exopolysaccharide as *P. ultramafica* STM10279^T^. Gonin et al. (2013) showed that *P. ultramafica* STM10279^T^ could solubilize phosphate, but there were no significant increases in phosphorus content when *T. comosa* was inoculated with *P. ultramafica* STM10279^T^. In our study, an improvement in growth was only observed after inoculation with the EPS of *P. ultramafica* STM10279^T^. In the aerial parts of these plants, there was a significantly higher concentration of phosphorus compared to both non-inoculated and *P. ultramafica* inoculated plants. The improvement in growth could be explained by this improvement in P concentration driven by the EPS. Yi et al. (2008) showed the great importance of EPS in the dissolution of tricalcium phosphate, apart from organic acids and protons. Therefore, it is likely to be indirectly involved in phosphate solubilization if a large amount of organic acid (e.g., citric acid) is produced by the PSB. Our experiment showed that *P. ultramafica* STM279^T^ EPS alone was capable of solubilizing phosphate. Contrary to the results of Yi et al. (2008), the addition of citric acid did not lead to an increase in phosphate solubilization. We found an antagonistic effect of citric acid on phosphate solubilization by EPS.

## 5 Conclusion

A study was conducted on the EPS extracted from *P. ultramafica* STM10279^T^, which revealed that it is a highly branched polymer that is similar to cepacian and EPS B from *P. kururiensis*. The presence of glucuronic acids in the EPS was confirmed, which could potentially help in reducing metal stress. Greenhouse experiments were conducted involving *T. comosa*. The experiments used EPS at the root level or inoculated with *P. ultramafica* STM10279^T^. The results showed that the EPS not only alleviates metal stress but also stimulates growth. This growth stimulation may be due to improved nutrition. Since *T. comosa* has little or no mycorrhizae, phosphate nutrition is crucial for its growth, especially in ultramafic soils. The *in vitro* study on the effect of *P. ultramafica* EPS on phosphate solubilization highlighted its ability to solubilize phosphate. This ability could explain the growth stimulation of *T. comosa* on ultramafic soil when amended with EPS. The findings indicate that EPS plays a crucial role in *P. ultramafica* STM10279^T^ in decreasing the metal levels in plants. However, additional research is required to ascertain whether the EPS generated by *P. ultramafica* is responsible for the observed impact on *T. comosa* growth. At the end of the experiment, we need to verify or quantify the presence of *P. ultramafica* in the roots or rhizosphere of *T. comosa*. Research will be conducted to understand how EPS chelates with metal ions. The aim is to use this knowledge to develop environmental metal remediation processes. The effectiveness of *P. ultramafica* STM10279^T^ EPS in reducing the harmful effects of metals on metal-sensitive plants should be studied. Our research indicates that amending *P. ultramafica* STM10279^T^ EPS directly to the soil is more effective than inoculating the plant with bacterial suspension for improving plant growth. Inoculating the soil with EPS has practical advantages such as easy storage and preparation. It is also an inert, non-living product, which reduces biological risks, making it marketable and safer for use in greenhouses. Direct inoculation of EPS could be a promising approach to improve plant growth and metal tolerance in ultramafic soils. It could also be a useful tool in global strategies for successfully restoring ultramafic areas after mining.

## Data availability statement

The original contributions presented in the study are included in the article/**Supplementary Material**. Further inquiries can be directed to the corresponding author.

## Author contributions

AB: Data curation, Formal analysis, Investigation, Methodology, Writing – original draft. GP: Formal analysis, Methodology, Writing – review & editing. HA: Supervision, Writing – review & editing, Validation. AL: Data curation, Formal analysis, Methodology, Writing – original draft. EC: Resources, Writing – review & editing. VM: Formal analysis, Methodology, Writing – review & editing. PJ: Writing – review & editing. PM: Writing – review & editing. VB-S: Funding acquisition, Supervision, Writing – review & editing. LG: Conceptualization, Funding acquisition, Project administration, Supervision, Writing – review & editing, Validation.
