# Supplementary material for: The cepacian-like exopolysaccharide of Paraburkholderia ultramafica STM10279T enhances growth and metal adaptation of Tetraria comosa on New Caledonian ultramafic soil
Source: Front Plant Sci. 2024 Jun 5;15:1349724. doi: 10.3389/fpls.2024.1349724 (PMC11188366; doi:10.3389/fpls.2024.1349724)
Supplement: Supplementary file 1 [file DataSheet_1.pdf]

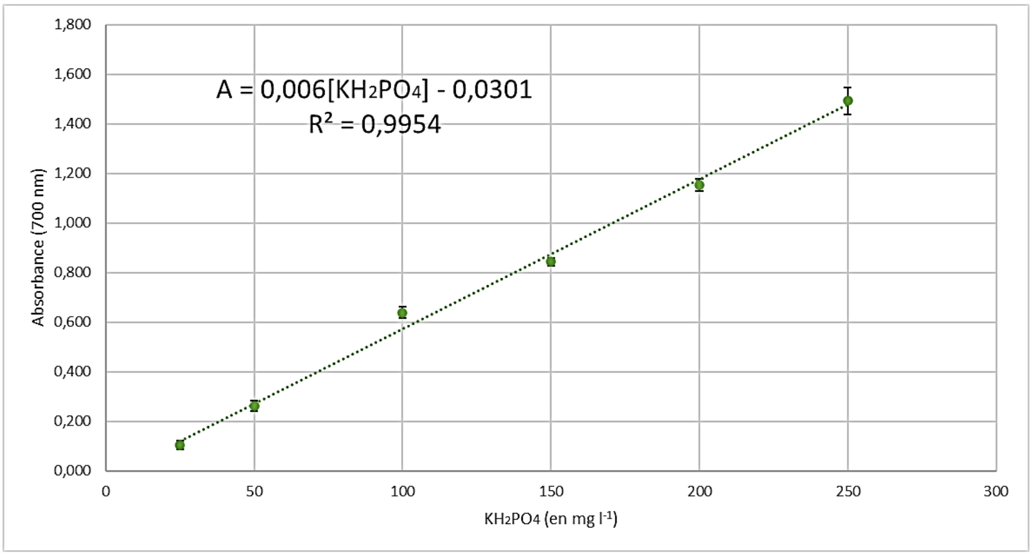

Fig S1. KH_2PO_4 standard curve (mean values with standard errors; n = 4)

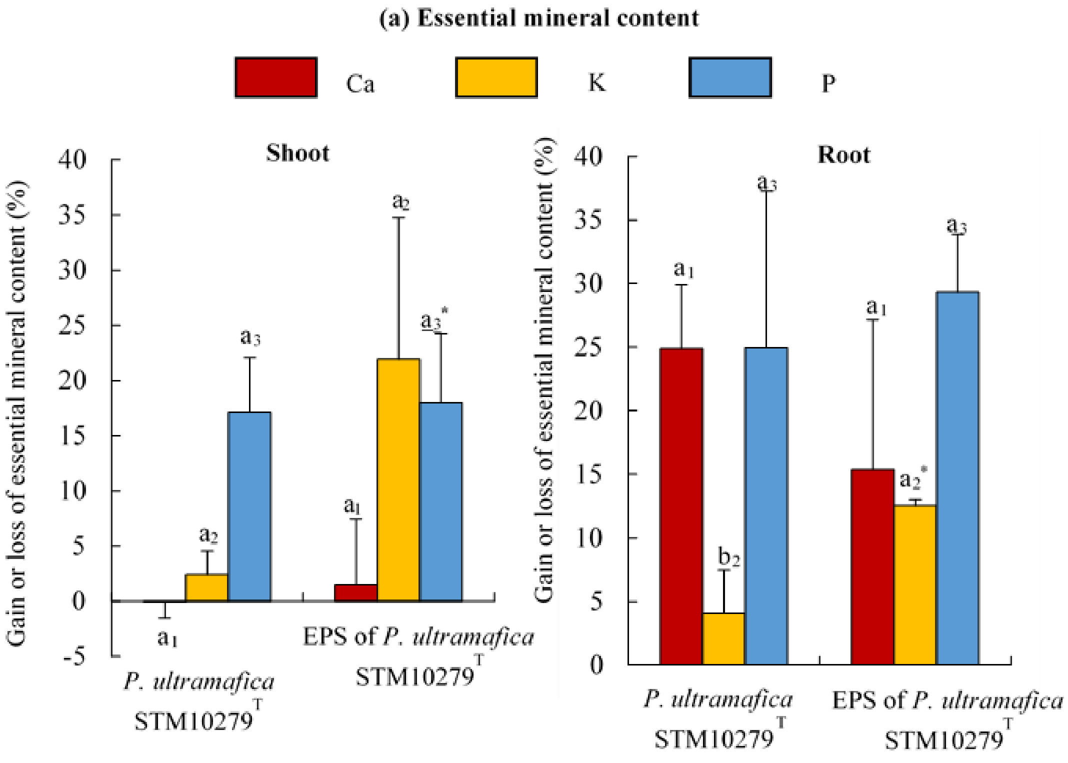

Fig. S2 Effect of inoculation on essential mineral content of *Tetraria comosa* grown on ultramafic substrate. Relative variations of element content in shoots and roots are expressed as % of element content in dry mass of tissues of inoculated plants compared with controls. Bars represent means, and error bars represent standard deviation of means (n = 3). Different letters above columns indicate significant differences at $P < 0.05$. The same coefficient to letters refers to one element analysis. The letters and coefficients with an asterix indicate significant differences with controls at $P < 0.05$.

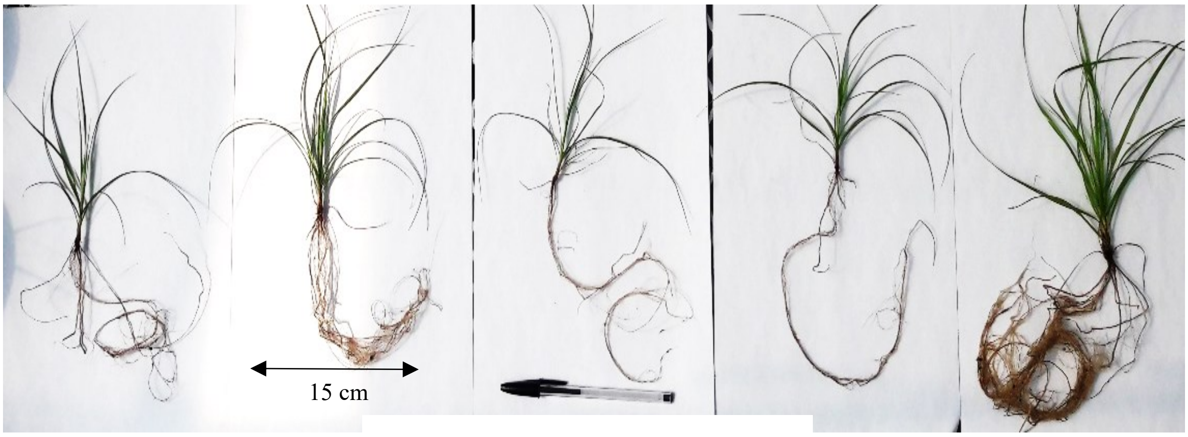

Control

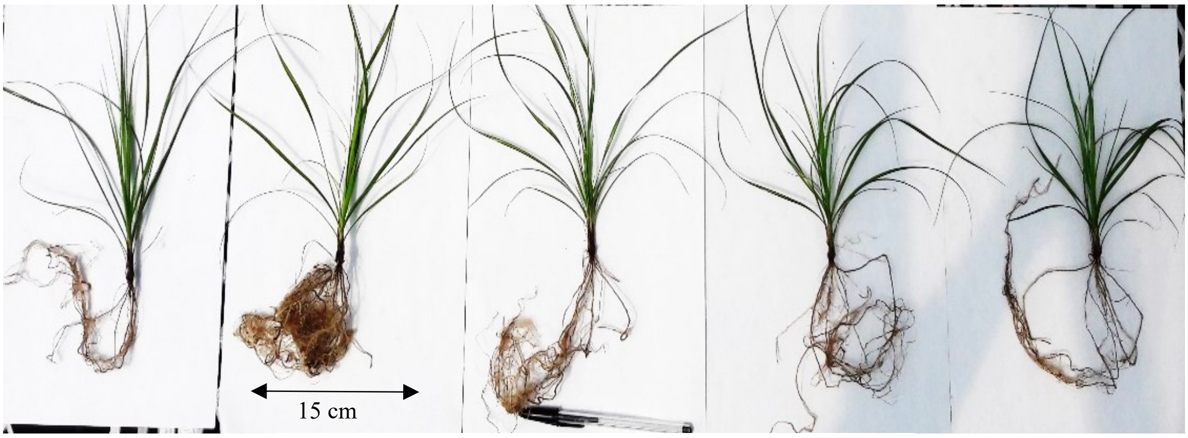

Paraburkholderia ultramafica STM10279^{T}

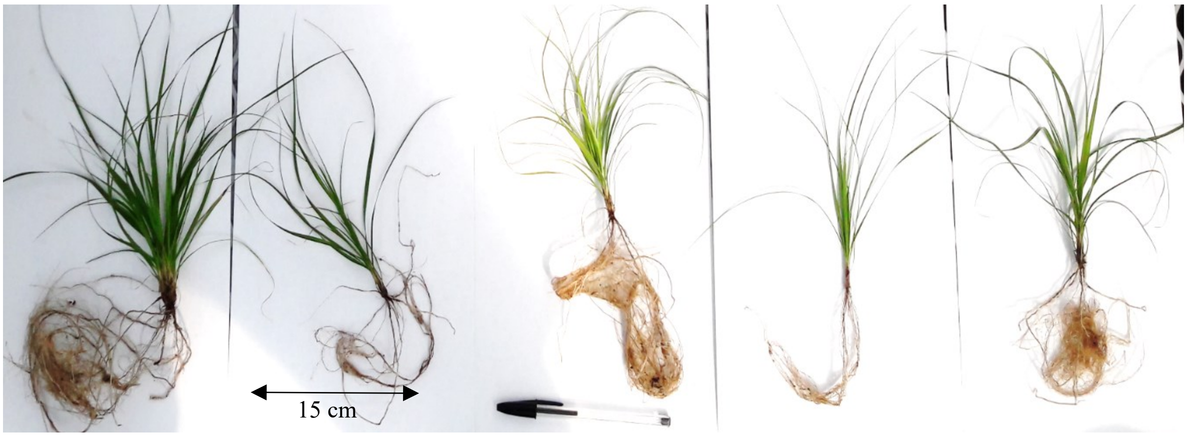

EPS of *Paraburkholderia ultramafica* STM10279^{T}

Fig S3. Photographic report of *Tetraria comosa* removed from pots after 8 months of grown in ultramafic soil (5 samples representative of the average of growth).

Supplementary Tables

Table S1 Major characteristics of *Paraburkholderia ultramafica* STM10279^T and the soil sampling site (Guentas et al., 2016; Bourles et al., 2020b).

	P. ultramafica STM10279^T	
**Sampling characteristics**		
Plant Host species	*Tetraria arundinacea*	
Site	Mont-Dore, Plum road<br>22°15'54"S; 166°35'57" E 22.26 S 166.59 E	
Altitude	534 m	
Geomorphological type*	Cambisol*	
Soil main characteristics		
pH (H_2O)	5.2	
	**Total (g Kg^{-1})**	**Extractable Elements (DTPA, mg Kg^{-1})**
N	1.63	
P (g Kg^{-1})	0.129	
Ca (g Kg^{-1})	0.46	
Mg (g Kg^{-1})	10.4	
Fe (g Kg^{-1})	396	95
Mn (g Kg^{-1})	2.7	76
Ni (g Kg^{-1})	3.9	162
Co (g Kg^{-1})	0.44	57
Cr (g Kg^{-1})	20.4	0.6
**Microbiological characteristics**		
Range temperature (°C)	20-32	
% NaCl	0-1	
pH optimum	5-6	
**Molecular characteristics**		
16S RNA seq EMBL	FR872407	
**Trace metal tolerance**	**IC_{50} (mM)**	
Ni	45.6 ± 5.3	
Co	3.9 ± 0.9	
Cr	0.5 ± 0.1	
Mn	32 ± 1.6	
**Plant Growth Promoting Traits**		
Phosphate solubilization	+	
Indole-3-acetic acid	+	
NH_3	+	
Siderophore	+	
ACC deaminase	+	

Note : ACC, 1-aminocyclopropane-1-carboxylate deaminase.

*Soil geomorphological definition according to FAO UNESCO and as reported by (Wulff et al, 2010)

Table S2 KH_2PO_4 standard curve

Concentration (mg l^{-1})	25	50	100	150	200	250
Absorbance (700 nm)	0.090	0.257	0.616	0.829	1.124	1.573
	0.087	0.235	0.622	0.828	1.143	1.455
	0.119	0.281	0.658	0.854	1.172	1.47
	0.121	0.278	0.656	0.859	1.173	1.471
**Mean**	0.104	0.263	0.638	0.843	1.153	1.492
**Standard deviation**	0.018	0.021	0.022	0.016	0.024	0.054

Table S3 Evaluation of the phosphorus solubilisation by the EPS of *P. ultramafica* STM10279^T expressed in mgl^{-1} of KH_2PO_4

Sample	EPS assay 1	EPS assay 2	EPS assay 3	Control
**Absorbance (700 nm)**	0.8	1.2	1.0	0.6
	0.8	1.2	1.0	0.6
	0.8	1.2	1.0	0.6
**Mean value**	0.8	1.2	1.0	0.6
**A_{assay} -$A_{control}$**	0.2	0.6	0.4	0.0
**Concentration P ($mg\ l^{-1}$) expressed in KH_2PO_4**	**46.5**	**109.7**	**68.5**	**0.0**
**Mean concentration ($mg\ l^{-1}$)**	**74.9**			
**Standard deviation**	**32.1**			

Table S4 Shoot and root biomasses of *Tetraria comosa* (mean ± SE n=15) as a function of various treatments.

Treatment	Shoot biomass (mg)	Root biomass (mg)
Control	243.3 ± 71.0b_1[†,‡]	116.7 ± 45.9a_2
P. ultramafica STM10279^T	391.0 ± 74.1ab_1	151.8 ± 40.3a_2
EPS of *P. ultramafica* STM10279^T	548.7 ± 116.7a_1	280.5 ± 79.8a_2

[†]Different letters in each column indicate significant differences at $P < 0.05$. [‡]Same coefficient to letters refers to one data analysis of one plant part (shoot or root).

Table S5 Mineral element content in shoot and root of *Tetraria comosa* (mean ± SE n = 3) as function of various treatment.

Plant Part	Treatment	Ca (g. Kg^{-1})	K (g. Kg^{-1})	P (mg. Kg^{-1})	Mg (g. Kg^{-1})	Na (g. Kg^{-1})
Shoot	Control	4.3 ± 0.3a_1[†,‡]	9.2 ± 0.2a_2	385 ± 27b_3	1.6 ± 0.1a_4	0.53 ± 0.15a_5
	P. ultramafica STM10279^T	4.3 ± 0.1a_1	9.4 ± 0.2a_2	451 ± 19ab_3	1.6 ± 0.0a_4	0.92 ± 0.09a_5
	EPS of *P. ultramafica* STM10279^T	4.4 ± 0.3a_1	11.2 ± 1.2a_2	454 ± 24a_3	1.5 ± 0.1a_4	0.36 ± 0.11a_5
Root	Control	1.6 ± 0.1a_1	6.0 ± 0.0b_2	260 ± 6a_3	1.5 ± 0.0c_4	0.92 ± 0.02b_5
	P. ultramafica STM10279^T	2.0 ± 0.1a_1	6.3 ± 0.2b_2	325 ± 32a_3	2.1 ± 0.1a_4	1.54 ± 0.14a_5
	EPS of *P. ultramafica* STM10279^T	1.8 ± 0.2a_1	6.8 ± 0.0a_2	337 ± 12a_3	1.7 ± 0.0b_4	0.59 ± 0.23b_5

[†]Different letters in each column indicate significant differences at $P < 0.05$. [‡]Same coefficient to letters refers to one data analysis of one element.

Table S6 Element uptake in shoot and root of *Tetraria comosa* (mean ± SE n = 3) as function of various treatment.

Plant Part	Treatment	Ca (mg. plant^{-1})	K (mg. plant^{-1})	P (µg. plant^{-1})	Mg (mg. plant^{-1})	Na (mg. plant^{-1})
Shoot	Control	1.04 ± 0.08c_1[†,‡]	2.24 ± 0.05c_2	94 ± 7c_3	0.39 ± 0.02c_4	0.13 ± 0.04b_5
	P. ultramafica STM10279^T	1.67 ± 0.02b_1	3.68 ± 0.08b_2	176 ± 8b_3	0.64 ± 0.01b_4	0.36 ± 0.03a_5
	EPS of *P. ultramafica* STM10279^T	2.39 ± 0.14a_1	6.15 ± 0.65a_2	249 ± 13a_3	0.80 ± 0.04a_4	0.20 ± 0.06ab_5
Root	Control	0.18 ± 0.01b_1	0.70 ± 0.00c_2	30 ± 1c_3	0.17 ± 0.00a_4	0.11 ± 0.00a_5
	P. ultramafica STM10279^T	0.30 ± 0.01b_1	0.95 ± 0.03b_2	49 ± 5b_3	0.32 ± 0.01a_4	0.23 ± 0.02a_5
	EPS of *P. ultramafica* STM10279^T	0.51 ± 0.05a_1	1.91 ± 0.01a_2	94 ± 3a_3	0.46 ± 0.01a_4	0.16 ± 0.07a_5

[†]Different letters in each column indicate significant differences at $P < 0.05$. [‡]Same coefficient to letters refers to one element uptake analysis.

Table S7 Trace metal content in shoot and root of *Tetraria comosa* (mean ± SE n = 3) as function of various treatment.

Plant Part	Treatment	Co	Cr	Fe	Mn	Ni
Shoot	Control	18.2 ± 2.0a_1[†,‡]	106 ± 9.8a_2	5.1 ± 0.6a_3	466 ± 8a_4	258 ± 5a_5
	P. ultramafica STM10279^T	13.0 ± 2.9a_1	90.4 ± 25.6a_2	3.7 ± 1.0a_3	517 ± 56a_4	231 ± 22a_5
	EPS of *P. ultramafica* STM10279^T	10.1 ± 0.9a_1	66.5 ± 1.1a_2	2.9 ± 0.1a_3	451 ± 17a_4	232 ± 38a_5
Root	Control	48.5 ± 6.0a_1	212.4 ± 31.0a_2	15.3 ± 2.0a_3	882 ± 86a_4	246 ± 32a_5
	P. ultramafica STM10279^T	82.6 ± 7.0a_1	417.3 ± 40.5a_2	27.5 ± 3.0a_3	1280 ± 100$_4$a	410 ± 35a_5
	EPS of *P. ultramafica* STM10279^T	76.5 ± 16.8a_1	388.9 ± 71.3a_2	24.5 ± 5.2a_3	1280 ± 238a_4	390 ± 84a_5

[†]Different letters in each column indicate significant differences at $P < 0.05$. [‡]Same coefficient to letters refers to one data analysis of one metal.
